# North Korean refugee health in South Korea (NORNS) study: study design and methods

**DOI:** 10.1186/1471-2458-12-172

**Published:** 2012-03-08

**Authors:** Yo Han Lee, Won Jin Lee, Yun Jeong Kim, Myong Jin Cho, Joo Hyung Kim, Yun Jeong Lee, Hee Young Kim, Dong Seop Choi, Sin Gon Kim, Courtland Robinson

**Affiliations:** 1 Department of Preventive Medicine, Korea University College of Medicine, Seoul, South Korea; 2 Division of Endocrinology and Metabolism, Department of Internal Medicine, Korea University Anam Hospital, Seoul, South Korea; 3 Department of Internal Medicine, Inje University Ilsan Paik Hospital, Goyang, South Korea; 4 Department of International Health, Center for Refugee and Disaster Response, Johns Hopkins Bloomberg School of Public Health, Baltimore, MD, USA

**Keywords:** North Korea, Refugee, Health

## Abstract

**Background:**

Understanding the health status of North Korean refugees (NKRs), and changes in health during the resettlement process, is important from both the humanitarian standpoint and the scientific perspective. The NOrth Korean Refugee health iN South Korea (NORNS) study aims to document the health status and health determinants of North Korean refugees, to observe various health outcomes as they occur while adapting to the westernized lifestyle of South Korea, and to explain the mechanisms of how health of migrants and refugees changes in the context of new environmental risks and opportunities.

**Methods:**

The NORNS study was composed of an initial survey and a follow-up survey 3.5 years apart. Participants were recruited voluntarily among those aged 30 or more living in Seoul. The survey consists of a health questionnaire and medical examination. The health questionnaire comprises the following six domains: 1) demographic and migration information 2) disease history, 3) mental health, 4) health-related lifestyle, 5) female reproductive health, and 6) sociocultural adaptation. The medical examination comprises anthropometric measurements, blood pressure and atherosclerosis, and various biochemical measurements. Prevalence of several diseases able to be diagnosed from the medical examination, the changes between the two surveys, and the association between the outcome and other measurements, such as length of stay and extent of adaptation in South Korea will be investigated. Furthermore, the outcome will be compared to a South Korean counterpart cohort to evaluate the relative health status of NKRs.

**Discussion:**

The NORNS study targeting adult NKRs in South Korea is a valuable study because various scales and medical measurements are employed for the first time. The results obtained from this study are expected to be utilized for developing a health policy for NKRs and North Korean people after unification. Additionally, since NKRs are an immigrant group who are the same race and have the same genetic characteristics as South Koreans, this study has the characteristics of a unique type of migrant health study.

## Background

North and South Korea, originally a united country, were divided during the Cold War and have since been situated in different politico-social environments. Since the late 1980s, and certainly in the 1990s, while South Korea has experienced drastic economic growth and modernization which led to an improved standard of living, North Korea has suffered great economic hardship and famine as a result of the collapse of the Communist bloc and consecutive natural disasters [1]. Since 1998, there has been a growing exodus of North Korean refugees (NKRs) seeking food or a better life, primarily into China as a first asylum country. Many of them are known to hope to permanently resettle in South Korea. In fact, the number of NKRs entering South Korea each year has increased steadily since 2000--indeed, resettlement numbers have averaged over 2,500 per year since 2008, and the total number of NKRs in South Korea was set to exceed 23,000 as of the end of 2011 [2].

As the number of NKRs residing in South Korea has increased, their settlement has become an important issue in South Korean society. Among various factors affecting the settlement of NKRs, their health remains one of the most critical [3]. Numerous studies have been conducted concerning the health of NKRs so far. These studies have shown that NKRs are vulnerable to depression, post-traumatic stress disorder, and other psychological problems in the process of defection and resettlement [4-8]. Their poor nutritional state before entering South Korea was also reported, and as a result, the average physiques of NKRs are reported to be much smaller than that of South Koreans, particularly in children and young adults [9-11]. A few studies on the general health of NKRs reported that they have more diseases than South Koreans, and the view on the health of these people was extremely negative [3,12].

However, most of the studies concerning the health of NKRs were limited to topics on mental health and nutrition/growth, and even the few studies on the general health of NKRs were based on a small number of subjects composed of their self-reports and self-ratings. Thus, a current comprehensive and objective understanding of the general health status of NKRs is limited. Moreover, since most of the studies have focused on health problems caused by living in North Korea and the countries of transit and temporary asylum, they do not highlight the immigrant health effects to westernized South Korea

The NOrth Korean Refugee health iN South Korea (NORNS) study, targeting adult NKRs living in South Korea, was launched with the following two objectives. First, the NORNS study intends to present the comprehensive health state of adult NKRs empirically, through a wide-ranging health questionnaire and a medical health examination of various items. Thus, the NORNS study is going to identify their current health status and health determinants. Second, the NORNS study is going to observe various health outcomes that are expected to occur while NKRs adapt to westernized South Korean society and to explain the mechanisms of these outcomes. Various studies on immigration research have revealed that environmental changes are important factors of the patterns and epidemiology of lifestyle-related and metabolic diseases [13-15]. The present article explains the significance of the NORNS study and also describes the study methodology.

## Methods

### Basic design

The NORNS study process is composed of two phases. The phase 1 study is a cross-sectional survey of NKR adults with a health questionnaire and medical examination on various items. The phase 2 study is a follow-up survey for same items 3.5 years after the phase 1 study.

### Subject recruitment

Participant eligibility was limited to NKRs aged 30 or more years living in Seoul. The greatest proportion of total NKRs in South Korea, 31.2%, resides in Seoul [2]. We placed notices on the Internet and by telephone once a month with the help of the Hana Center, a representative welfare center supported by the government for assisting NKRs who have settled in South Korea, that a health questionnaire survey and medical examinations would be conducted every month for NKRs above 30 years old living in Seoul; and applicants applied to participate voluntarily. In this way, a total of 550 subjects have been recruited from October 2008 until December 2011

### Survey process

**Figure 1:**
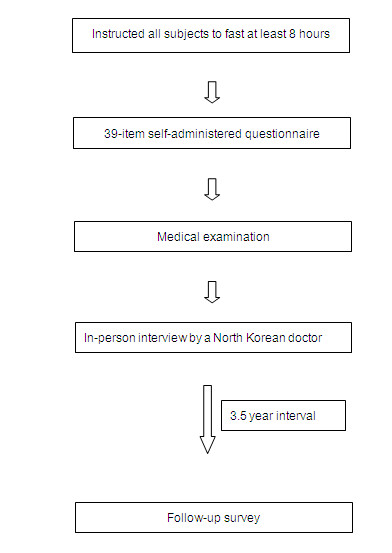
**The whole process of NORNS study**.

Figure 1 depicts the whole process of the survey. The phase 1 survey has been conducted at the Korea University Anam Hospital located in northeastern Seoul once a month. All subjects were notified that they should fast from midnight of the day before the survey. The questionnaire survey was conducted for about 30 minutes, and those who completed the questionnaire were given medical examinations. Additionally, we tried to enhance the quality and completeness of the questionnaire surveys by having the subjects have a one-to-one talk with a doctor who escaped from North Korea, on the basis of the questionnaire contents.

### Questionnaire

We developed a 42-item health questionnaire on the basis of the existing questionnaires aimed at NKRs and the questionnaire of the Korea National Health and Nutrition Examination Survey (KNHANES) [16] and was composed of the following six domains: 1) demographic and migration information? 2) disease history, 3) mental health status, 4) health-related lifestyles, 5) female reproductive health, and 6) sociocultural adaptation (Table 1).

**Table 1:** Measurements of this study

Measurement	Description
**Questionnaire**	

Demographic characteristics	Sex, age, education level in NK, main job in both NK and SK, marital status in both NK and SK, current income level, resident area in NK, date of escape, date of arrival at SK

Disease history	Diseases diagnosed by medical doctor in both NK and SK

Mental health	Depression, psychological stress, suicide trial, suicide ideation

Health-related life style	Smoking, alcohol drinking, exercise, health check-up taking, nutritional supplements intake

Women-specific conditions	Menstruation status, pregnancy and delivery history

**Medical examination**	

Anthropometric measurements	Height, weight, waist circumference, hip circumference and body composition analysis

Blood pressure	

Test for atherosclerosis	Pulse wave velocity at both brachial and ankle arteries

Biochemical measurement	Complete blood cell count, liver and renal function, glucose, insulin, thyroid hormones, lipid profile, other metabolic tests

NK, North Korea; SK, South Korea.

### Demographic and migration information

General socio-demographic indicators, such as occupation, level of income and education, marriage status in both North and South Korea, and age and sex were obtained. For assessing migration indices of the subjects, the time of escape from North Korea and arrival in South Korea were obtained so that the length of stay in South Korea and in third countries can be calculated. Information on the subjects' residential areas in North Korea and countries of transit and temporary asylum after defection were also included.

### Disease history

Past disease history and current status of 32 typical diseases in the cardiovascular system, musculoskeletal system, gastroenterological system, respiratory system, communicable diseases, and cancers diagnosed by doctors in South and North Korea were obtained.

### Mental health

For the systematic assessment of the NKRs' mental health, which was found to be very poor by previous studies, the following instruments were used. For identifying depression, the Korean edition of the Center for Epidemiological Studies Depression Scale, a 20-item instrument which frequently serves as a depression screening tool in epidemiological research, was used [17,18]. For identifying psychosocial stress, an 18-item psychosocial well-being index-short form composed of a 4-point scale, which has been developed and validated in South Korea, was used [19]. Information on suicide ideation and suicide attempts during the preceding 1 year was also obtained.

### Lifestyle factors

Health-related lifestyles, such as smoking, alcohol consumption, and exercise, were measured. Detailed smoking histories, including age at first cigarette, packs per day, and number of smoking and smoke-free years, were obtained. Alcohol consumption habits like intake amount and frequency during the preceding 12 months were assessed. We also determined whether or not subjects participated in regular exercise, in addition to the duration of such exercise.

### Female reproductive health

We tried to examine the reproductive state and history of female subjects, since female NKRs accounted for three-quarters of total NKRs in South Korea, and their gynecological and obstetric health has been reported as very poor. The questionnaire has items on histories of menstrual state, pregnancy, and birth that are identical to those of KNHANES.

### Sociocultural adaptation scale

Searl and Ward [20] developed a scale to evaluate psychological and sociocultural forms of adjustment during the process of cross-cultural transitions. The scale was transformed into a new 29-item scale adjusted to situations NKRs have faced, and items such as 'making friends,' 'using the transport system,' and 'talking about yourself with others' are included in the new adaptation scale.

### Medical examination

The basic medical examination, which was conducted for all subjects, consists of anthropometric measurement, blood pressure and atherosclerosis examination, biochemical measurement, thyroid sonogram, and bone densitometry (Table 1).

### Anthropometric measurement

Height, weight, and body mass index were recorded. Body fat mass and body fat percentage (BFP) were measured by bioelectrical impedance analysis (Inbody 720; Biospace, Seoul, Korea). The BFP measurement that is derived from a bioelectrical impedance analysis is less accurate than measurements obtained by dual-energy X-ray absorptiometry. However, this method of estimating body composition has become increasingly popular because it is easy to use, non-invasive, relatively inexpensive, provides an accurate method for the assessment of body composition, and can be performed across a wide range of subjects with regard to age and body shape [21]. Waist circumference, measured in triplicate at the midpoint between the lower rib and the iliac crest, and hip circumference, measured in triplicate at the point yielding the maximum circumference over the buttocks, were also included.

### Blood pressure and atherosclerosis check

Systolic blood pressure (SBP) and diastolic blood pressure (DBP) were measured by an automatic blood pressure monitor (TM-2655P; Biospace, Japan) on the arm of a seated subject who had rested in a sitting position for 10 minutes before the measurement. The TM-2655 device achieved British Hypertension Society grade A/A and therefore can be recommended for blood pressure measurement in an adult population [22]. The mean of the two SBP and DBP measurements at least 5 minutes apart were used for the analysis. After a subject had rested in a supine position for 5 minutes, brachial-ankle pulse wave velocity (baPWV) and ankle-brachial index were measured using a volume-plethymographic apparatus (model VP 1000; Colin, Komaki, Japan). This instrument simultaneously records baPWV and the brachial and ankle blood pressures on the left and right sides. baPWV is considered a risk marker [23] and prognostic predictor of atherosclerosis [24]. baPWV is a newly developed device using a volume-rendering method. Due to its technical simplicity and short sampling time, baPWV is more appropriate for screening a large population than previous methods. The validity and reliability of the automated device for measuring baPWV have been established previously [25].

### Biochemical measurements

Blood samples were drawn after an overnight fast. Serum total cholesterol, triglycerides, high-density lipoprotein cholesterol, low-density lipoprotein cholesterol, uric acid, and liver enzyme levels were determined by enzymatic methods with a chemistry analyzer (TBA 200-FR; Toshiba, Japan). Plasma glucose was measured by the glucose oxidase method. High-sensitivity C-reactive protein was determined by latex turbidimetry (TBA 200-FR; Toshiba). Serum insulin was measured with an insulin radioimmunoassay kit (Diasource, Nivelles, Belgium), which had a reactivity of less than 0.2% to human proinsulin. Insulin resistance was estimated using the homeostasis model of assessment, calculated as fasting glucose (mmol/L) × fasting insulin (mU/L)/22.5. Microproteinuria was measured with turbidimetry (TBA 40-FR; Toshiba). Various adiopocytokine levels and thyroid functions were also measured.

### In-person interview by a North Korean doctor

We employed a defected North Korean doctor who has the same background and experience as general NKRs. We had him have one-to-one interviews with all subjects and verify the important items of the questionnaire with his cultural and linguistic knowledge, whereby we tried to enhance the validity and completeness of the subjects' reports.

### Follow-up survey

The phase 2 follow-up survey started April 2012, which is 3.5 years after the first subject group was examined with the phase 1 survey. Subject recruitment for the phase 2 survey also will be aided by the Hana Center which has the contact information of the subjects, and only for voluntarily applied ones. Survey process and measurements are almost identical to the phase 1 survey methods, but additional examination will be provided.

### Participants

The number of participants recruited and surveyed from December 2008 to December 2010 was a total of 440, and female participants accounted for 75% (n = 330; Table 2). The average age was 42.6 for females and 46.9 for males, but subjects in their thirties accounted for the greatest portion. In both females and males, about half of the total subjects have education levels of high school or less and careers in manual work, and more than 70% of them were married. On average, males resided in South Korea for 41.1 months and the females for 36.6 months. A difference in length of stay in third countries was noted between the sexes. While more than half of the male subjects entered South Korea less than 1 year after defection from North Korea, it had taken 4 years or more for about half of the female subjects to enter South Korea, and the average duration for females in third countries was 49.5 months, more than double that of the males.

**Table 2:** Demographic characteristics of study subjects by sex

	Men (n = 110)		Women (n = 330)	
	**n**	**%**	**n**	**%**

**Age**				

Mean (SD)	46.9 (12.4)		42.6 (10.6)	

30-39	38	34.6	157	47.6

40-49	34	30.9	98	29.7

50-59	14	12.7	37	11.2

60-	24	21.8	38	11.5

**Education in North Korea**				

High school or less	52	51.5	170	54.8

Higher specialized school	20	19.8	83	26.8

College or more	29	28.7	57	18.4

**Occupation in North Korea**				

Manual worker	50	47.6	134	42.0

Farmer	2	1.9	16	5.0

Office worker	18	17.1	44	13.8

Soldier	9	8.6	6	1.9

Teacher	4	3.8	52	4.7

Student	0	0.0	8	2.5

Professional	19	18.1	70	21.9

Unemployed	3	2.9	26	8.2

**Marital status in North Korea**				

Married	85	80.2	211	69.9

Unmarried	21	19.8	91	30.1

**Marital status in North Korea**				

Married	85	80.2	211	69.9

Unmarried	21	19.8	91	30.1

**Residence in North Korea**				

Urban	64	66.0	184	64.8

Rural	33	34.0	100	35.2

**Length of stay in South Korea Korea(months)**				

Mean (SD)	41.0 (35.8)		36.6 (30.0)	

< 12	27	24.8	91	28.1

12-24	18	16.5	45	13.9

24-48	25	22.9	87	26.9

≥ 48	39	35.8	101	31.2

**Length of stay in third countries country(months)**				

Mean (SD)	23.2 (31.5)		49.5 (43.4)	

< 12	62	56.9	95	29.5

12-24	14	12.8	22	6.8

24-48	13	11.9	46	14.3

≥ 48	20	18.4	159	49.4

### Statistical analysis

#### Descriptive analysis

Baseline health characteristics of the subjects will be identified from descriptive analysis of questionnaire survey and medical examination. From the analysis of questionnaire survey results, distribution of subjects for most variables measured will be described by sex and age groups. From the analysis of medical examination results, average levels of measurements and empirical prevalence of diseases, such as hypertension, diabetes, dyslipidemia, obesity, and metabolic syndrome which can be diagnosed by the medical examination, will be obtained. All expression levels will be described as number (%) or mean (standard deviation).

#### Comparative analysis

The baseline health results of the subjects will be compared to those of South Korean counterparts to evaluate the relative health state of NKRs. The raw data of KNHANES, a nationwide survey for the South Korean people, will be used as the representative sample. For ensuring comparability between the two groups, we will extract two to fourfold samples of South Korean people who are age- and gender-matched to the North Korean subjects. A *x*2-test or Fisher's exact test and two-sample *t*-test will be used for the comparison of categorical variables, such as disease prevalence and numeric variables, respectively. For comparison of anthropometric measurements such as height and weight, the gap between the two groups will be calculated with units of measurement.

#### Statistical analysis

Multiple regression and multiple logistic regression analyses will be performed to investigate the association among various measurements. In particular, the association between disease prevalence and reported characteristics, such as demographic indices, lifestyles, mental health state, and migration index, will be investigated. Changes in disease prevalence and biochemical measurements according to the length of stay in the third countries or South Korea will be evaluated with a trend test. A statistical method for a longitudinal or panel study, such as a generalized estimating equation or pooled cross-sectional time-series analysis, will be used for evaluating changes between two measurements.

### Ethics

Approval of the study was obtained from the Institutional Review Board of Korea University Medical Center (approval number: ED08023), and all participants provided written informed consent.

## Discussion

The NORNS study is a research study targeting adult NKRs in South Korea and intends to show empirically their overall health state, health determinants, and changes in health patterns. It is a more comprehensive study than previous ones, in which more subjects and more measurement items were used compared to the existing research, and practical measurement was employed for the first time.

It is important to investigate and understand the overall health status of NKRs living in South Korea for the following reasons. First, from a humanitarian standpoint, it is critical to support their health institutionally because, as a vulnerable minority group, they have many health problems and difficulties in the adaptation to their new lives. Second, despite factors that make people who migrate different from people who don't, they can serve as useful proxies for North Korean people who are seldom known and unable to be contacted. It is possible to assess the health level of North Korean people through evaluating NKRs. Last but not least, findings of changes in their health and disease pattern while adapting to westernized and urbanized South Korean society will provide valuable scientific knowledge on the pathophysiology of non-communicable diseases and insight on the health changes in North Korean people which will occur after unification.

The results achieved from this study are expected to be utilized as references in establishing policies on refugees in South Korea and as a database on health-related policies of North Koreans after the unification of the two Koreas.

NKRs are a unique immigrant group that has the same genetic characteristics as South Koreans but has been exposed to two-step changes, a different environment due to the division of two Koreas and then also resettlement in South Korea. That is, the NORNS study can suggest a unique type of migration study, while ordinary migration studies investigate differences between a group that did not migrate and a group that migrated to different nations. In addition, the study group will provide significant data on correlations between changes of environment and diseases and on the thrift phenotype hypothesis [26].

The NORNS are the Goddesses of Destiny in Norse mythology, who rule the past, the present, and the future and shape the lives of humans from their first day to their last. The authors decided to name this study 'NORNS' in the hope that the destiny of the two Koreas, though divided at present, will be unified in the near future. The health of North Korean refugees in South Korea can be an important key to shaping a more positive destiny for the Korean people.

## Abbreviations

baPWV: brachial-ankle pulse wave velocity; BFP: body fat percentage; DBP: diastolic blood pressure; KNHANES: Korea National Health and Nutrition Examination Survey; NKR: North Korean refugee; NORS: North Korean refugee health in South Korea; SBP: systolic blood pressure.

## Competing interests

The authors declare that they have no competing interests.

## Authors' contributions

YHL drafted and wrote the manuscript. SGK obtained funding and supervised the entire process of research and writing. All authors made substantial contributions to conception, design, and revision of the manuscript. All authors read and approved the final manuscript.

## Pre-publication history

The pre-publication history for this paper can be accessed here:

http://www.biomedcentral.com/1471-2458/12/172/prepub
